# Supplementary material for: Engineered heart muscle allografts for heart repair in primates and humans
Source: Nature. 2025 Jan 29;639(8054):503–11. doi: 10.1038/s41586-024-08463-0 (PMC11903342; doi:10.1038/s41586-024-08463-0)
Supplement: Supplementary file 1 — Supplementary Methods (flow cytometry gating strategies), Supplementary Tables 1–6, Supplementary Notes and Clinical Trial Protocol. [file 41586_2024_8463_MOESM1_ESM.pdf]

nature portfolio

https://doi.org/10.1038/s41586-024-08463-0

Supplementary information

Engineered heart muscle allografts for heart repair in primates and humans

In the format provided by the authors and unedited

Engineered heart muscle allografts for heart repair in primates and humans

Supplementary Information

Ahmad-Fawad Jebran[1,2,*], Tim Seidler[2,3,4*], Malte Tiburcy[2,5,*], Maria Daskalaki[2,6], Ingo Kutschka[1,2], Buntaro Fujita[7,8], Stephan Ensminger[7,8], Felix Bremmer[2,9], Amir Moussavi[2,10], Huaxiao Yang[11,12], Xulei Qin[11,12], Sophie Mißbach[2,13], Charis Drummer[2,6], Hassina Baraki[1,2], Susann Boretius[2,10], Christopher Hasenauer[14], Tobias Nette[14], Johannes Kowallick[2,14], Christian O. Ritter[2,14], Joachim Lotz[2,14], Michael Didié[2,3], Mathias Mietsch[2,13], Tim Meyer[2,5], George Kensah[1,2], Dennis Krüger[15], Md Sadman Sakib[15], Lalit Kaurani[15], Andre Fischer[2,15,16,17], Ralf Dressel[2,18], Ignacio Rodriguez-Polo[2,6], Michael Stauske[2,6], Sebastian Diecke[19], Kerstin Maetz-Rensing[20], Eva Gruber-Dujardin[20], Martina Bleyer[20], Beatrix Petersen[2,20], Christian Roos[21], Liye Zhang[21], Lutz Walter[2,21], Silke Kaulfuß[22], Gökhan Yigit[2,22], Bernd Wollnik[2,17,22], Elif Levent[2,5], Berit Roshani[23], Christiane Stahl-Henning[23], Philipp Ströbel[9], Tobias Legler[2,24], Joachim Riggert[2,24,†], Christian Hellenkamp[2,3], Jens-Uwe Voigt[25], Gerd Hasenfuß[2,3], Rabea Hinkel[2,13], Joseph C. Wu[11,12], Rüdiger Behr[2,6], Wolfram-Hubertus Zimmermann[2,5,17,26,27]

*equal contribution

[1]Clinic for Cardiothoracic Surgery, University Medical Center Göttingen, Germany; [2]German Center for Cardiovascular Research (DZHK), partner site Lower Saxony, Germany; [3]Clinic for Cardiology and Pneumology, University Medical Center Göttingen, Germany; [4]Department of Cardiology, Campus Kerckhoff of the Justus-Liebig-Universität Gießen, Kerckhoff-Clinic, Bad Nauheim, Germany; [5]Institute of Pharmacology and Toxicology, University Medical Center Göttingen, Germany; [6]Platform Degenerative Diseases, German Primate Center – Leibniz Institute for Primate Research, Göttingen, Germany; [7]Clinic for Cardiac and Thoracic Vascular Surgery, University Medical Center Schleswig Holstein, Campus Lübeck, Lübeck, Germany; [8]German Center for Cardiovascular Research (DZHK), partner site Hamburg/Kiel/Lübeck, Germany; [9]Institute of Pathology, University Medical Center Göttingen, Germany; [10]Functional Imaging Laboratory, German Primate Center, Göttingen, Germany; [11]Stanford Cardiovascular Institute and [12]Department of Medicine, Division of Cardiovascular Medicine, Stanford University School of Medicine, Stanford, CA, USA; [13]Laboratory Animal Science Unit, German Primate Center – Leibniz Institute for Primate Research, Göttingen, Germany; [14]Institute of Diagnostic and Interventional Radiology, University Medical Center Göttingen, Germany; [15]Department for Epigenetics and Systems Medicine in Neurodegenerative Diseases, German Center for Neurodegenerative Diseases (DZNE), Göttingen, Germany; [16]Department of Psychiatry and Psychotherapy, University Medical Center, Göttingen, Germany; [17]Cluster of Excellence "Multiscale Bioimaging: from Molecular Machines to Networks of Excitable Cells" (MBExC), University of Göttingen, Germany; [18]Institute of Cellular and Molecular Immunology, University Medical Center Göttingen, Germany; [19]Pluripotent Stem Cells Platform, Max Delbrück Center for Molecular Medicine in the Helmholtz Association (MDC), Berlin, Germany; [20]Pathology Unit, German Primate Center – Leibniz Institute for Primate Research, Göttingen, Germany; [21]Primate Genetics Laboratory, German Primate Center – Leibniz Institute for Primate Research, Göttingen, Germany; [22]Institute of Human Genetics, University Medical Center Göttingen, Germany; [23]Unit of Infection Models, German Primate Center – Leibniz Institute for Primate Research, Göttingen, Germany; [24]Department of Transfusion Medicine, University Medical Center Göttingen, Germany; [25]Department of Cardiovascular Diseases, University Hospital Leuven, Leuven, Belgium; [26]German Center for Neurodegenerative Diseases (DZNE), Göttingen, Germany; [27]Fraunhofer Institute for Translational Medicine and Pharmacology (ITMP), Göttingen, Germany

Corresponding author: Wolfram-Hubertus Zimmermann, M.D.
Institute of Pharmacology and Toxicology
University Medical Center Göttingen - Georg-August-University
Robert-Koch-Str. 40; 37075 Göttingen, Germany
Tel: +49-551-39-65781 Fax: +49-551-39-5699
Email: w.zimmmermann@med.uni-goettingen.de

Supplementary Methods

Supplementary Method 1: Flow Cytometry Gating Strategies.

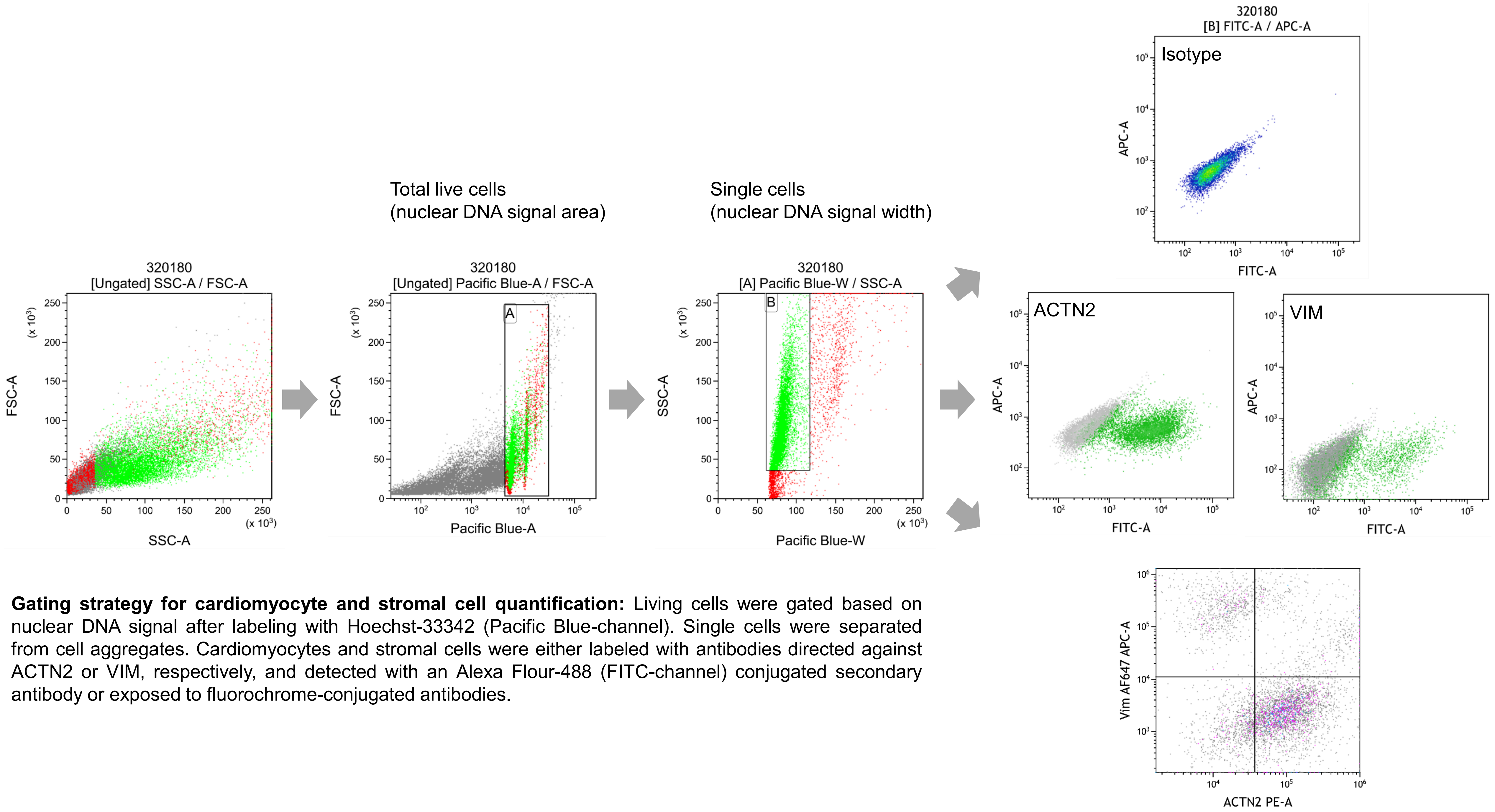

Gating strategy for cardiomyocyte and stromal cell quantification: Living cells were gated based on nuclear DNA signal after labeling with Hoechst-33342 (Pacific Blue-channel). Single cells were separated from cell aggregates. Cardiomyocytes and stromal cells were either labeled with antibodies directed against ACTN2 or VIM, respectively, and detected with an Alexa Flour-488 (FITC-channel) conjugated secondary antibody or exposed to fluorochrome-conjugated antibodies.

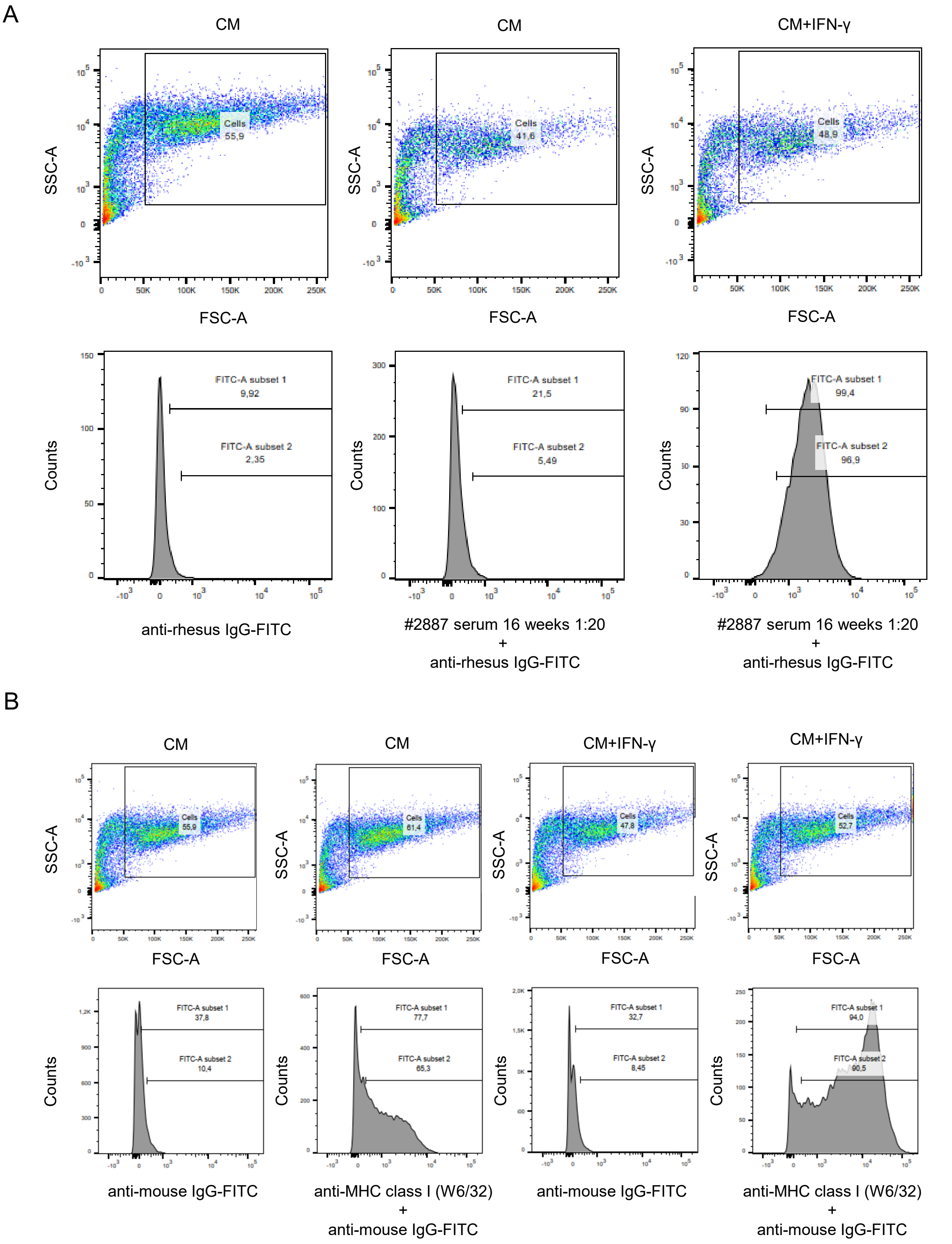

Gating strategy for donor specific antibody (DSA) detection: (**A**) Cardiomyocytes (CM) not stimulated or stimulated with IFN-γ for 48 h were gated based on FSC-A and SSC-A parameters to exclude debris and not incubated or incubated with 1:20 diluted sera obtained from #2887 16 weeks after EHM implantation and after withdrawal of immunosuppression. 20.000 events were measured. A FITC-labeled anti-rhesus IgG antibody detected antibodies in the sera bound to the CMs. In addition to the mean fluorescence intensity, the proportion of stained CMs has been determined using the second marker (FITC-A subset 2). (**B**) The expression of MHC class I molecules on the CMs used in this experiment has been determined in parallel using the W6/32 antibody and a FITC-labeled secondary antibody against mouse IgG. Antibodies that display a selective reactivity to IFN-γ-stimulated CMs presumably include DSAs to MHC class I molecules.

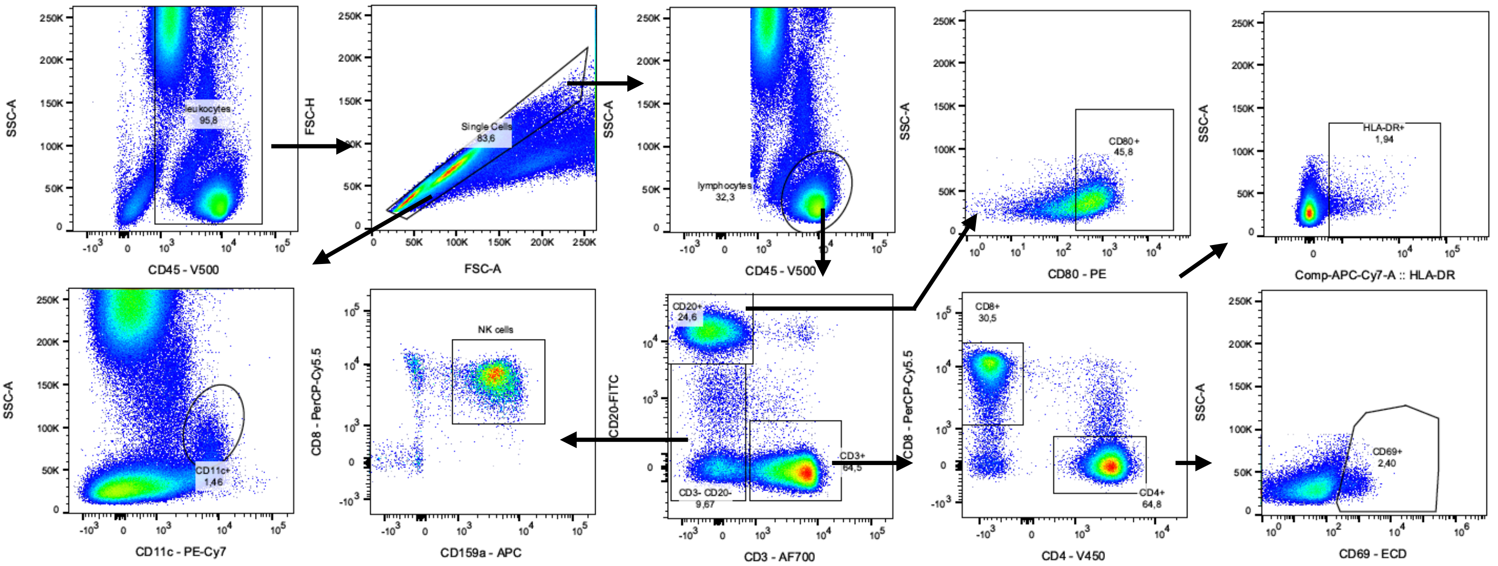

Gating strategy peripheral blood mononuclear cells: Leukocytes were gated based on CD45 expression versus SSC-A. Following exclusion of douplets either CD11c+ cells or CD45+ lymphocytes were further gated. T and B cells were distinguished based on CD3 versus CD20 expression. T cells were further divided into CD4+ and CD8+ T cells. NK cells were identified as CD3- CD20-/CD8+ CD159a+ cells. Activation of immune cells was assessed by analyzing CD80 expression on B cells and CD11c+ cells as well CD69 and HLA-DR expression on total CD3+, CD4+, CD8+ T cells and NK cells.

Supplementary Tables:

Supplementary Table 1: Overview of Rhesus macaque iPSC lines used in the study. CNPRC: California National Primate Research Center, Davis, US; DPZ: Deutsches Primatenzentrum (German Primate Center), Göttingen, Germany.

Supplementary Table 2: Basic contractility data of human and Rhesus EHM.

Supplementary Table 3: Overview of Rhesus macaques in Cohorts 1-3.

Supplementary Table 4: MRI data summary (Cohorts 1 and 2 – Healthy Model).

Supplementary Table 5: MRI data summary (Cohort 3 – Heart Failure Model).

Supplementary Table 6: Antibodies used in the study. RTU: ready to use. *cross-reactive with Rhesus macaque mitochondria / not cross-reactive with rat mitochondria

Supplementary Table 1

Origin	Origin	Reprogramming Method	Implant	Reference
Rhesus iPSC 43110-4	Skin fibroblasts (CNPRC)	CytoTune-iPSC Kit (Thermo Fisher)	allograft	Zhao et al. 2018
Rhesus iPSC DPZ_iRH25.B1	Skin fibroblasts (DPZ)	CytoTune-iPSC Kit (Thermo Fisher)	autograft	unpublished
Rhesus iPSC DPZ_iRH23.1	Skin fibroblasts (DPZ)	Episomal Vectors	autograft	Stauske et al. 2020
Rhesus iPSC DPZ_iRH34.1	Skin fibroblasts (DPZ)	Episomal Vectors	allograft	Stauske et al. 2020

Supplementary Table 2

iPSC-line		TC1133	43110-4	DPZ_iRH34.1	#2483	#2500
Species		Human	Rhesus	Rhesus	Rhesus	Rhesus
EHM sample number		16	12	10	4	4
Spontaneous Beating Rate (bpm)		51±3	82±5	93±4	123±7	93±6
@1.5 Hz electrical field stimulation	max. FOC (mN)	1.1±0.1	0.64±0.13	0.26±0.04	0.28±0.02	0.04±0.01
	Resting Tension (RT in mN)	0.6±0.08	0.36±0.05	0.65±0.07	0.63±0.11	0.46±0.09
	FOC/RT	2.2±0.3	1.8±0.3	0.44±0.07	0.48±0.08	0.09±0.04
	Contraction Time (to 90% in ms)	140±4	112±3	104±3	64±3	68±6
	Relaxation Time (to 50% in ms)	114±4	99±3	88±3	65±2	86±7

Supplementary Table 3

Cohort 1 (3 months follow-up)

Animal#	Age	Gender	BW	Implant	Dose	Immunesuppression
2444	9.1 yrs	male	13.7 kg	allograft§	1x EHM (34,960,000 CM / 5,040,000 StC)[i]	Tacrolimus + Methylprednisolone
2529	8.5 yrs	male	12.4 kg	allograft§	1x EHM (33,240,000 CM / 6,760,000 StC)[i]	Tacrolimus
2441	10.2 yrs	male	8.3 kg	allograft§	1x EHM (36,960,000 CM / 3,040,000 StC)[i]	Tacrolimus + Methylprednisolone
2616	7.5 yrs	female	7.0 kg	allograft§	1x EHM (36,960,000 CM / 3,040,000 StC)[i]	Tacrolimus
2520	9.1 yrs	male	9.3 kg	allograft§	1x EHM (32,240,000 CM[ii] / 7,760,000 StC)[i]	Tacrolimus + Methylprednisolone
2551	11.3 yrs	female	7.6 kg	allograft§	1x EHM (32,240,000 CM[ii] / 7,760,000 StC)[i]	Tacrolimus + Methylprednisolone
2483	9.9 yrs	female	6.2 kg	autograft	1x EHM (30,744,000 CM[ii] / 9,256,000 StC)	none

Cohort 2 (6 months follow-up)

Animal#	Age	Gender	BW	Implant	Dose	Immunesuppression
2506	9.6 yrs	female	10.2 kg	allograft§	5x EHM (188,800,000 CM[ii] / 11,200,000 StC)[i]	Tacrolimus + Methylprednisolone
2500	10 yrs	female	7.6 kg	autograft	5x EHM (125,400,000 CM / 74,600,000 StC)[i]	none
2869	5 yrs	male	7.3 kg	allograft§	5x EHM (129,060,000 CM[ii] / 70,940,000 StC)	Tacrolimus + Methylprednisolone[iii]
2887	4.9 yrs	male	6.9 kg	allograft§	5x EHM (156,600,000 CM[ii] / 43,400,000 StC)	Tacrolimus + Methylprednisolone[iii]
2909	4.8 yrs	male	7.8 kg	allograft§	5x EHM (156,600,000 CM[ii] / 43,400,000 StC)	Tacrolimus + Methylprednisolone
2913	4.7 yrs	male	6.5 kg	allograft§	5x EHM (112,860,000 CM[ii] / 87,140,000 StC)	Tacrolimus + Methylprednisolone
2915	4.8 yrs	male	7.6 kg	allograft§	5x EHM (112,860,000 CM[ii] / 87,140,000 StC)	Ciclosporin + Methylprednisolone

Cohort 3 (6 months follow-up)

Animal#	Age[iv]	Gender	BW[v]	Implant	Dose	Immunosuppression
2651	9 yrs	male	13.6 kg	no graft	not applicable (Control)	no immunosuppression
2650	9 yrs	male	11.0 kg	no graft	not applicable (Control)	Tacrolimus + Methylprednisolone
2750	8 yrs	male	11.0 kg	no graft	not applicable (Control)	Tacrolimus + Methylprednisolone
2907	5.9 yrs	male	10.6 kg	allograft§	2x EHM (62,352,000 CM / 17,648,000 StC)	Tacrolimus + Methylprednisolone
2911	5.1 yrs	male	9.1 kg	no graft	death during reperfusion	not applicable
16299	9.2 yrs	male	9.4 kg	no graft	not applicable (Control)	Tacrolimus + Methylprednisolone
2762	8.2 yrs	female	9.5 kg	no graft	not applicable (Control)	no immunosuppression
16356	8.3 yrs	male	7.2 kg	allograft§	2x EHM (69,408,000 CM[ii] / 10,592,000 StC)	Tacrolimus + Methylprednisolone
2735	8.2 yrs	female	8.2 kg	no graft	not applicable (Control)	Tacrolimus + Methylprednisolone
16721	7.1 yrs	male	8.3 kg	allograft§	2x EHM (66,096,000 CM / 13,904,000 StC)	Tacrolimus + Methylprednisolone
2771	7.3 yrs	female	6.6 kg	no graft	SCD 1 week post-MI	not applicable
2819	7.3 yrs	male	9.8 kg	allograft§	5x EHM (161,460,000 CM[ii] / 38,540,000 StC)	Tacrolimus + Methylprednisolone
15301	9.6 yrs	female	7.5 kg	no graft	SCD 1 week post-MI	not applicable
2739	8.1 yrs	male	11.1 kg	allograft§	5x EHM (171,000,000 CM[ii] / 29,000,000 StC)[vi]	Tacrolimus + Methylprednisolone
2884	6.6 yrs	male	9.7 kg	allograft§§	5x EHM[vii] (158,400,000 CM[ii] / 41,600,000 StC)	Tacrolimus + Methylprednisolone
2719	7.9 yrs	male	10.1 kg	no graft	death during reperfusion	not applicable
2868	6.7 yrs	male	6.6 kg	no graft	not applicable (Control)	no immunosuppression
15266	10.1 yrs	female	6.7 kg	no graft	death during reperfusion	not applicable
15389	n.a.	female	6.0 kg	no graft	excluded (low body weight)	not applicable
16441	7.8 yrs	male	8.2 kg	allograft§§	5x EHM[vii] (166,770,000 CM[ii] / 33,230,000 StC)	Tacrolimus + Methylprednisolone

Allografts constructed from Rhesus iPSC 43110-0§ or DPZ_iRH34.1§§

[i] cells from cardiomocyte (CM) differentiation without addition of separately prepared stromal cells (StCs; number = per flow cytometry identified non-CM content)
[ii] use of cyropreserved cardiomyocytes (CM) for EHM formulation (stromal cells [StCs] were in all cases used after cryopreservation)
[iii] withdrawal after 3 months to induce rejection
[iv] at the end of the study
[v] at the time of EHM implantation / last weight recorded in animals that died prematurely or were excluded from the study
[vi] death upon weaning from aneasthesia
[vii] 5x EHM fusion

Supplementary Table 4

	Condition (pre vs. post EHM)	Heart rate (bpm)	EF (%)	EDV (mL)	ESV (mL)	TWThd (mm)	TWThs (mm)	TWThF (%)	CLWThd (mm)	CLWThs (mm)	CLWThF (%)
	1x EHM (n=7)	103±6 vs. 107±6	57±1 vs. 58±1	16±2 vs. 15±2	7.1±1.0 vs. 6.3±1.0	3.1±0.1 vs. 4.6±0.3***	6.5±0.2 vs. 7.7±0.2**	118±10 vs. 78±8**	3.4±0.2 vs. 3.9±0.2	5.9±0.4 vs. 6.0±0.4	78±14 vs. 57±6
	5x EHM (n=7)	107±3 vs. 102±4	59±1 vs. 63±1*	15±1 vs. 15±1	6.2±0.4 vs. 5.4±0.4	3.1±0.3 vs. 7.6±0.8***	7.1±0.4 vs. 11.6±1.0***	137±4 vs. 59±8***	3.3±0.1 vs. 3.7±0.2	5.5±0.2 vs. 6.0±0.2	73±7 vs. 65±5
1x EHM	Tac + MP (n=4)	101±9 vs. 108±10	56±2 vs. 58±2	16±2 vs. 14±2	7.1±1.0 vs. 6.0±1.0	3.3±0.2 vs. 4.5±0.3*	6.7±0.4 vs. 7.9±0.2*	110±10 vs. 86±10	3.4±0.3 vs. 3.9±0.3	6.5±0.4 vs. 6.2±0.4	96±19 vs. 63±6
	Tac (n=2)	110 vs. 108	59 vs. 58	19 vs. 17	7.8 vs. 7.3	3.0 vs. 5.3	6.2 vs. 8.0	109 vs. 58	3.6 vs. 4.2	5.7 vs. 6.6	66 vs. 62
	No ISP (autograft; n=1)	95 vs. 101	60 vs. 59	14 vs. 14	5.5 vs. 5.9	2.6 vs. 3.6	6.2 vs. 6.6	171 vs. 90	2.8 vs. 3.3	3.7 vs. 4.0	34 vs. 24
5x EHM	Tac + MP (n=3)	115±1 vs. 105±6	59±1 vs. 65±1*	15±2 vs. 14±2	6.0±0.8 vs. 4.8±0.9	3.4±0.5 vs. 9.0±1**	7.6±0.8 vs. 13.4±1.0*	136±7 vs. 55±11**	3.1±0.2 vs. 3.6±0.4	5.3±0.5 vs. 5.6±0.4	75±15 vs. 59±8
	Tac + MP 3 months (n=2)	98 vs. 91	59 vs. 63	17 vs. 16	6.7 vs. 5.9	3.3 vs. 6.7	7.3 vs. 10.9	127 vs. 76	3.6 vs. 3.9	5.9 vs. 6.3	71 vs. 68
	Cycl + MP (n=1)	110 vs. 107	56 vs. 59	15 vs. 15	6.5 vs. 6.3	2.7 vs. 6.3	6.6 vs. 9.7	150 vs. 59	3.0 vs. 3.5	5.5 vs. 6.3	85 vs. 85
	No ISP (autograft; n=1)	99 vs. 109	62 vs. 61	15 vs. 14	5.7 vs. 5.6	2.4 vs. 6.9	5.8 vs. 9.4	146 vs. 39	3.4 vs. 3.9	5.3 vs. 6.0	54 vs. 57

Data presented as mean ± SEM / n = macaques

Data points: pre implant (average of two baseline recordings on the day of and ~44 days before EHM implantation)
post implant (average of three (1x EHM Cohort 1) or four (5x EHM Cohort 2) recordings at 4, 8, 12, and 24 weeks after EHM implantation)

*P < 0.05; **P < 0.01; ***P < 0.001 - two-tailed, unpaired t-test pre vs post (EHM) implant

Supplementay Table 5

	Heart Rate (bpm)				Enddiastolic Volume (mL)				Endsystolic Volume (mL)				Ejection Fraction (%)			
Condition	No ISP	ISP	2x EHM	5x EHM	No ISP	ISP	2x EHM	5x EHM	No ISP	ISP	2x EHM	5x EHM	No ISP	ISP	2x EHM	5x EHM
Pre-MI	99±3	*75±8*	88±4	88±6	15.3±3.0	*17.3±0.7*	15.4±0.7	14.8±1.4	6.5±1.6	*7.5±0.2*	6.3±0.6	6.1±0.9	58±4	*56±1*	59±2	59±4
Post-MI / Pre-Implant	97±2	82±6	82±6	97±6	17.7±4.9	19.6±1.8	19.5±1.2	17.6±1.7	9.0±2.8	9.8±0.9	11.3±1.2*	9.7±1.7*	50±2	50±2	43±3*	46±6*
1 month post implant	103±2	88±5	87±6	98±1	17.3±4.9	16.8±1.4	15.7±1.6[§]	13.4±1.4[§]	8.7±2.7	7.9±0.8[§]	9.1±1.2[§]	6.8±1.8	51±1	52±4	43±3	52±10
2 months post implant	101±7	85±3	89±5	99±4	18.6±4.7	16.6±1.5	16.1±2.6	13.2±0.8	9.6±2.8	8.1±0.7[§]	9.1±1.7	6.3±1.4	50±2	50±4	44±5	53±8
3 months post implant	106±7	87±4	86±9	100±8	17.9±4.8	16.1±1.2	14.7±0.8	12.9±1.5	9.8±2.8	7.9±0.6	8.1±0.8	6.3±1.8	46±1	51±4	45±2	53±8
6 months post implant	101±8	86±6	83±7	94±6	18.1±4.6	14.8±1.1	14.5±0.5[§]	12.1±0.9[§]	9.6±2.4	7.5±0.5	8.4±0.6	5.8±0.9	47±4	49±2	42±3	52±6

	Target Wall Thickness in Diastole (mm)				Target Wall Thickness in Systole (mm)				Target Wall Thickening Fraction (%)			
Condition	No ISP	ISP	2x EHM	5x EHM	No ISP	ISP	2x EHM	5x EHM	No ISP	ISP	2x EHM	5x EHM
Pre-MI	3.6±0.4	*3.4±0.45*	3.3±0.6	3.7±0.9	4.8±0.5	*5.0±0.5*	5.2±1.0	5.4±1.1	41±7	*48±7*	58±4	53±6
Post-MI / Pre-Implant	2.4±0.2	2.1±0.3	2.0±0.1	3.5±0.6	2.5±0.2*	2.2±0.3*	1.9±0.2*	3.6±0.7	6±1*	3±3*	3±1*	3±4*
1 month post implant	2.5±0.2	1.9±0.2	3.6±0.7	6.0±0.8[§]	2.5±0.2	2.1±0.3	3.8±0.8	6.9±0.6[§]	1±1	8±3	6±2	16±6
2 months post implant	2.3±0.1	2.0±0.3	3.7±0.9	5.4±0.4[§]	2.7±0.2	2.2±0.4	4.2±1.0	6.2±0.2	15±8	5±4	16±2	16±5
3 months post implant	2.2±0.4	2.4±0.5	3.5±0.6	5.4±0.5[§]	2.3±0.4	2.5±0.6	4.3±0.9	6.1±0.1	7±3	2±5	20±7	17±9
6 months post implant	2.2±0.4	2.2±0.3	3.5±0.6	5.3±0.5[§]	2.3±0.4	2.3±0.4	3.8±0.8	6.0±0.2	6±4	3±6	8±5	15±9

	Contralateral Wall Thickness in Diastole (mm)				Contralateral Wall Thickness in Systole (mm)				Contralateral Wall Thickening Fraction (%)			
Condition	No ISP	ISP	2x EHM	5x EHM	No ISP	ISP	2x EHM	5x EHM	No ISP	ISP	2x EHM	5x EHM
Pre-MI	3.4±0.5	*3.6±0.6*	3.7±0.2	4.0±0.1	5.9±0.9	*5.5±0.7*	5.9±0.4	6.4±0.4	77±14	*56±15*	60±8	64±8
Post-MI / Pre-Implant	4.2±0.3*	4.1±0.3*	3.8±0.2	4.1±0.2	6.2±0.6	5.8±0.3	5.8±0.3	6.4±0.2	54±1	44±8	55±2	63±1
1 month post implant	3.7±0.8	3.9±0.4	4.5±0.5	5.1±0.1	6.2±1.0	5.8±0.6	6.4±0.1	7.4±0.1	72±14	54±2	52±15	53±2
2 months post implant	4.1±0.6	3.8±0.2	4.5±0.5	4.6±0.1	5.9±0.7	6.4±0.4	6.9±0.2	6.8±0.3	53±6	74±4	61±14	54±6
3 months post implant	3.7±0.8	4.1±0.5	4.7±0.6	4.9±0.2	6.2±1.1	6.4±0.4	6.6±0.4	7.4±0.4	74±4	63±8	45±10	59±7
6 months post implant	3.9±0.6	4.4±0.4	4.5±0.6	4.4±0.2	5.9±0.6	6.9±0.5	7.1±0.7	7.0±0.2	59±10	61±13	59±7	67±4

Data presented as mean ± SEM / n = macaques

Data points:
- Pre-MI (1 time point 175±6 days before EHM implantation)
- Post-MI / Pre-Implant (20±3 days before EHM implantation)
- 1 months post implant (27±1 days after EHM implantation)
- 2 months post implant (55±1 days after EHM implantation)
- 3 months post implant (84±1 days after EHM implantation)
- 6 months post implant (167±1 days after EHM implantation)

No ISP group: n = 3
ISP group: n = 4 (*n=3*)
2x EHM group: n = 3
5x EHM group: n = 3

*P < 0.05 Pre-MI vs. Post-MI / Pre-Implant - Mixed-effect model with Šídák's multiple comparisons test
[§]P < 0.05 vs. Post-MI / Pre-Implant - Two-Way-ANOVA with Dunnett's multiple comparison test with Geisser-Greenhouse correction

Supplementary Table 6

Primary Antibodies

Use	Epitope	Species	Dilution	Vendor	Catalog #
Immune fluorescence (IF)	Cardiac Troponin T	Rabbit	1:200	Abcam	ab45932
	Human-mitochondria (FITC-conjugated)*	Mouse	1:100	Millipore	MAB1273A4
	Oct3/4	Mouse	1:100	Santa Cruz	sc-5279
	Nanog	Rabbit	1:100	Santa Cruz	sc-33760
	Sox2	Rabbit	1:100	Cell Signaling	mAb #3579
	ACTN2 (clone EA-53)	Mouse	1:1,000	Sigma	A7811
	Rhesus IgG (FITC-conjugated)	Mouse	1:100	Southern Biotech	4700-02
Flow Cytometry (FC)	ACTN2 (clone EA-53)	Mouse	1:4,000	Sigma	A7811
	ACTN2 (PE-conjugated)	Mouse	1:1,000	Miltenyi Biotec	130-106-937
	VIM	Rabbit	1:1,000	abcam	ab92547
	VIM (AF647-conjugated)	Mouse	1:1,000	Biolegend	677807
	CD3 (AF700-conjugated)	Mouse	1:50	BD	557917
	CD4 (V450-conjugated)	Mouse	1:1,000	BD	560811
	CD8 (PerCP-Cy5.5-conjugated)	Mouse	1:25	BioLegend	301032
	CD11c (PE-Cy7-conjugated)	Mouse	1:25	BioLegend	301608
	CD20 (FITC-conjugated)	Mouse	1:25	BioLegend	302304
	CD45 (V500-conjugated)	Mouse	1:50	BD	561489
	CD69 (ECD-conjugated)	Mouse	1:100	Beckman Coulter	6607110
	CD80 (PE-conjugated)	Mouse	1:25	BD	557227
	HLA-DR (APC-Cy7conjugated)	Mouse	1:50	BioLegend	307618
	CD159a (APC-conjugated)	Mouse	1:50	Beckman Coulter	A60797
	pan-HLA class I (clone W6/32)	Mouse	1:200	BioLegend	311402
	REA293 / rh(c) IgG1 (PE-conjugated)	Human	1:1,000	Miltenyi Biotec	130-118-347
	MOPC-173 / IgG2a (AF647-conjugated)	Mouse	1:500	BioLegend	400240
Immunohistochemistry (IHC)	ACTN2 (clone EA-53)	Mouse	1:500	Sigma	A7811
	Desmin (clone D33)	Mouse	RTU	Dako	IR 60661-2
	OCT3/4 (clone N1NK9)	Mouse	RTU	Dako	IR 09261-2
	vWF (polyclonal)	Rabbit	RTU	Dako	IR 52761-2
	Ki67 (clone MIB-1)	Mouse	RTU	Dako	IR 62661-2
	Slow skeletal troponin I (clone OTI8H8)	Mouse	1:100	Novus Biologicals	NBP2-46170
	Cardiac troponin I (polyclonal)	Rabbit	1:200	abcam	ab47003
	Myosin Light Chain 2a (clone S58-205)	Mouse	1:250	BD Pharmigen	565496
	Myosin Light Chain 2v (polyconal)	Rabbit	1:250	Proteintech	10906-1-AP
	N-cadherin (polyclonal)	Rabbit	1:50	Santa Cruz	sc7939
	Connexin 43 (polyclonal)	Rabbit	1:500	abcam	ab217676
	CD3 (polyclonal)	Rabbit	RTU	Dako	GA50361-2
	CD20 (clone L26)	Mouse	RTU	Dako	IR60461-2
	CD56 (clone 123C3)	Mouse	RTU	Dako	IR62861-1
	CD57 (clone TB01)	Mouse	RTU	Dako	GA64761-2
	CD68 (clone PG-M1)	Mouse	RTU	Dako	IR61361-2
	TCRα/β (clone 8A3)	Mouse	1:50	Invitrogen	TCR1151
	TCRγ/δ (clone H-41)	Mouse	RTU	Master diagnostica	MAD-780QD-3
	Cleaved Caspase 3	Rabbit	1:200	Cell Signal Tech	9664S

Secondary Antibodies

Use Epitope	Species	Dilution	Vendor	Catalog #
Anti-rabbit IgG (Alexa Fluor 633-conjugated)	Goat	1:200	Thermo Fisher	A-21071
Anti-mouse IgG (Alexa Fluor 594-conjugated)	Goat	1:200	Thermo Fisher	A-11032
Anti-rabbit IgG (Alexa Fluor 594-conjugated)	Goat	1:200	Thermo Fisher	A-11012
Anti-mouse IgG (Alexa Fluor 488-conjugated)	Goat	1:1,000	Thermo Fisher	A-11029
Anti-mouse IgG (FITC-conjugated)	Goat	1:200	Dianova	115-095-062
Anti-rabbit (horseradish peroxidase-conjugated)	Goat	RTU	Dako	K800921-2
Anti-mouse (horseradish peroxidase-conjugated)	Goat	RTU	Dako	K802121-2

Supplementary Notes:

(1) Clarification of snRNseq analyses presented in Extended Data Figure 1.

Panels of transcripts used to identify and distinguish cardiomyocytes, stromal cells, pluripotent cells, and osteochondral cells by single nucleus RNA-sequencing.

Cardiomyocyte Panel (curated list according to Tiburcy et al. 2017):

ACACB, ACTCA1, ACTC1, ACTN2, ANKRD1, APOA1, APOBEC2, APOE, ATP1A2, ATP1B1, BMP5, C7, CAMK2B, CASQ2, CAVIN4, CCDC141, CGNL1, CHD7, CKM, CKMT2, CLIC5, CNN1, COL15A1, COL23A1, COL4A5, CPE, CRIP2, CRYAB, CSRP3, CYP2J2, DES, DMD, DTNA, EEF1A2, ENO3, EPHA4, FABP3, FHOD3, FMNL3, FZD3, GABRB1, GATM, GNG7, HAND2, HCN4, HOOK1, HOPX, HSPB3, HSPB7, IGFBP2, ITGA7, ITGB1BP2, KCNJ12, KCNQ5, KCNT1, KRT8, LAPTM4B, LDB3, LMOD2, MB, MDK, MEF2A ,MLF1, MLEP, MOGAT1, MTUS1, MYADML2, MYBPC3, MYH6, MYH7, MYL2, MYL3, MYL4, MYL7, MYOCD, MYOM1, MYOZ2, NCAM1, NEBL, NPNT, NPPA, NPPB, NREP, PDK1, PLN, PLXDC1, POPDC2, PPARGC1B, PPM1L, PPP1R1C, PPP1R3A, PPP1R9A, PREX2, PRKAA2, PROX1, PYGM, RAB6B, RASGRP2, RBFOX1, RBPMS2, RCAN2, RGS5, RGS6, RNASE1, RORB, RRAGD, RYR2, SCN5A, SGCA, SH3BGR, SLC25A4, SLC8A1, SLC8A1-AS1, SMPX, SMYD1, SNTA1, SPINT2, TBX20, TBX5, TECRL, THBS4, TMEM176B, TMEM71, TNNI1, TNNI3, TNNT2, TPM1, TRIM24, TTN, YPEL2

Stromal cell panel (curated list according to Tiburcy et al. 2017):

ABCG2, ACTG2, ADAM33, ADAMTS2, ADAMTS5, ADH1B, ADM, ALDH1A3, ALDH3B1, ALPL, ANKRD29, ANPEP, ANXA1, ANXA2, APOD, ARHGAP22, ARHGEF28, ARSJ, ATOH8, ATP8B1, AXL, BDNF, BHMT2, BST1, C8orf4, CAMK2N1, CCBE1, CCDC144A, CCDC36, CCL11, CCND1, CCR1, CD109, CD248, CD34, CD44, CD9, CDH19, CDKN2C, CFH, CLDN11, CLEC11A, CLEC14A, CLEC3B, CLMP, COMP, COL12A1, COL1A1, COL8A1, COLEC10, COLEC12, COTL1, CPED1, CPXM2, CRIM1, CRLF1, CSF2RB, CTSF, CTSK, CTSS, CXCL1, CYP1B1, DAB2, DCN, DDX3Y, DIO2, DKK2, DLL4, DOCK10, DPP4, DRAM1, EBF3, EDN1, EGFR, EHF, ELN, EMP1, ENG, ENPP2, ERAP2, F3, FAM162B, FAM180A, FAM20C, FAP, FBLN1, FBLN5, FGF2, FMO2, FN1, FRMD6, GIMAP2, GPR176, GPR68, HIST1H1A, HIST1H2BB, HLA-DMB, HMGA1, HMOX1, HPCAL1, HSPA2, IL6, IL7R, ITPR3, L1CAM, LAMA3, LAYN, LGALS1, LIMA1, LOX, LOXL3, LPAR1, LPXN, LRRK1, LRRN4CL, LTBP2, LTF, MAMDC2, MEOX2, MFAP5, MGLL, MKRN3, MLPH, MME, MMP2,

MR1, MRGPRF, MT1X, MT2A, MUSK, MYC, MYO10, NAALADL1, NBL1, NEGR1, NEK10, NFASC, NGF, NKAPL, NOV, NQO1, NR4A2, NT5E, NTN4, OAS2, OLFML1, OSR2, PAMR1, PCDH18, PCOLCE, PEG3, PID1, PLAU, POSTN, PRDM1, PRELP, PRR16, PTGER4, PTGS1, PTX3, RASSF2, RGCC, RGMB, RND3, S100A4, S100A6, SAMD9L, SERPINE1, SERPINE2, SFRP4, SH2D4A, SH3GL2, SHISA3, SLC14A1, SLC16A4, SLC1A1, SLC37A2, SLCO4A1, SLFN11, SLFN5, SPESP1, SPHK1, SQRDL, SRGN, STC1, STC2, SVEP1, SYNE3, SYTL2, TAGLN2, TBC1D2, TCF21, TDRD1, THBS1, TNC, TNFRSF11B, TRPA1, USP9Y, UTY, VASN, VEGFC, VGLL3, VIM, VIT, WNT5B, ZFY, ZNF662

Osteochondral cell panel:
ACAN, COMP, EPYC, MGP, OGN, PHOSPHO1, SHOX2, SOX5

Pluripotent cell panel
ALPL, DNMT3B, GDF3, LIN28A, NANOG, POU5F1, TERT, ZFP42

Remaining unidentified cells were individually identified as cardiomyocytes, mesodermal stromal cells or mesendodermal cells using the following rules:

Cardiomyocyte identification by expression of:
1. TNNT2 amongst the top 1,000 detected transcripts
2. ACTC1 amongst the top 1,000 detected transcripts
3. ACTN2 amongst the top 1,000 detected transcripts
4. TTN amongst the top 100 detected transcripts
5. RYR2 amongst the top 100 detected transcripts
6. DMD amongst the top 100 detected transcripts

Extended Data Figure 1A (i): 8 of 17 initially unidentified cells
Extended Data Figure 1A (ii): 285 of 401 initially unidentified cells
Extended Data Figure 1A (iii): 74 of 87 initially unidentified cells
Extended Data Figure 1B (i): 0 of 40 initially unidentified cells
Extended Data Figure 1B (ii): all cells annotated
Extended Data Figure 1C (i): 504 of 573 initially unidentified cells
Extended Data Figure 1C (ii): 52 of 77 initially unidentified cells

Mesodermal stromal cell identification by expression of:

1. FN1 amongst the top 1,000 detected transcripts
2. COL1A1 amongst the top 1,000 detected transcripts
3. COL1A2 amongst the top 1,000 detected transcripts
4. CALD1 amongst the top 1,000 detected transcripts

Extended Data Figure 1A (i): 2 of 17 initially unidentified cells

Extended Data Figure 1A (ii): 94 of 401 initially unidentified cells

Extended Data Figure 1A (iii): 13 of 87 initially unidentified cells

Extended Data Figure 1B (i): 39 of 40 initially unidentified cells

Extended Data Figure 1B (ii): all cells annotated

Extended Data Figure 1C (i): 53 of 573 initially unidentified cells

Extended Data Figure 1C (ii): 17 of 77 initially unidentified cells

Mesendodermal cell identification by expression of:

1. MEF2A amongst the top 1,000 detected transcripts
2. AFP amongst the top 1,000 detected transcripts

Extended Data Figure 1A (i): 7 of 17 initially unidentified cells

Extended Data Figure 1A (ii): 12 of 401 initially unidentified cells

Extended Data Figure 1A (iii): 0 of 87 initially unidentified cells

Extended Data Figure 1B (i): 0 of 40 initially unidentified cells

Extended Data Figure 1B (ii): all cells annotated

Extended Data Figure 1C (i): 12 of 573 initially unidentified cells

Extended Data Figure 1C (ii): 5 of 77 initially unidentified cells

The procedure resulted in the annotation of >99% of the cells in the different differentiations:

Extended Data Figure 1A (i): all 3,264 (0%) cells annotated

Extended Data Figure 1A (ii): 10 of 3,874 (0.34%) cells not annotated

Extended Data Figure 1A (iii): all 3,098 (0%) cells annotated

Extended Data Figure 1B (i): 1 of 2,251 (0.04%) cells not annotated

Extended Data Figure 1B (ii): all 3,022 (0%) cells annotated

Extended Data Figure 1C (i): 4 of 3,630 (0.11%) cells not annotated

Extended Data Figure 1C (ii): 3 of 1,339 (0.22%) cells not annotated

(2) Clinical Research Information

Provided according to Nature editorial policies @:

https://www.nature.com/nature/editorial-policies/clinical-research

Registration:

BioVAT-HF-DZHK20 is registered under EudraCT No. 2019-000885-39 (EU CT No. 2024-515708-38-01) and ClinicalTrials.gov ID NCT04396899

Study Protocol:

The most recent version (V6) of the Clinical Trial Protocol Synopsis is provided. We are referring to a single case from BioVAT-HF, which was subjected to heart transplantation.

Interim Analysis:

Does not apply – we are reporting a case from BioVAT-HF (the only patient from this trial that was heart transplanted as of today) – interim analysis of BioVAT-HF is anticipated for the second half of 2025.

Reporting Guidelines:

We report a case from the BioVAT-HF-DZHK Phase I/II clinical trial. Information as to the origin of the data (from BioVAT-HF-DZHK20 EudraCT No. 2019-000885-39 [EU CT No. 2024-515708-38-01] / ClinicalTrials.gov ID NCT04396899) is included. The patient history with a limited set of clinical data is provided in Extended Data Figure 9. The full clinical data will be reported at the as to the study protocol predetermined endpoint.

Data Sharing:

A Data Availability Statement (DAS) is included in lines 316-319 of the main manuscript.

Competing interests:

A Competing Interests statement is included in lines 946-950 of main manuscript file.

DZHK
DEUTSCHES ZENTRUM FÜR
HERZ-KREISLAUF-FORSCHUNG E.V.

Clinical Trial Protocol

Safety and Efficacy of Induced Pluripotent Stem Cell-derived Engineered Human Myocardium as Biological Ventricular Assist Tissue in Terminal Heart Failure

BioVAT-HF

Engineered Human Myocardium (EHM) in patients with terminal heart failure

**EU CT No.**	2024-515708-38-01
**FOMA-ID**	02289
**ClinicalTrials.gov ID**	NCT04396899
**Protocol Version**	V 6.0, 13.08.2024
**Therapeutic area**	Terminal heart failure
**Revision chronology, if applicable**	Version 5.2<br>Version 5.1<br>Version 5.0<br>Version 4.2<br>Version 3.0<br>Version 2.0
**Development Phase**	Phase I/II
**Sponsor**	University Medical Center Göttingen<br>represented by the Head of the Clinical Trials Unit<br>Von-Bar-Str. 37075 Göttingen, GERMANY
**Coordinating Scientist**	Prof. Dr. Wolfram-Hubertus Zimmermann<br>University Medical Center Göttingen<br>Robert-Koch-Str. 40<br>37075 Göttingen

This Clinical Trial Protocol contains confidential information. Circulation of this material to individuals who are not involved in the carrying out of the study or any kind of publication requires the approval of the sponsor. These limitations similarly relate to all confidential information and data which will be obtained in the future.

Synopsis

**TITLE OF TRIAL**	Safety and Efficacy of Induced Pluripotent Stem Cell-derived Engineered Human Myocardium as Biological Ventricular Assist Tissue in Terminal Heart Failure
**SHORT TITLE**	BioVAT-HF
**EU CT NO**	2024-515708-38-01
**FOMA-ID**	02289
**HEALTH CONDITION STUDIED**	Terminal heart failure
**PHASE**	Phase I/II
**OBJECTIVE(S)**	Primary objective:<br>• to assess safety and efficacy of Engineered Human Myocardium (EHM) in patients with terminal heart failure (HFrEF EF ≤35%) with or without RV dysfunction (TAPSE <16 mm)<br>Secondary objective:<br>• to assess effects of EHM-grafts on disease-specific events and symptoms

<table>
<tr>
<td>TREATMENT(S)</td>
<td>
<u>Experimental intervention/Index test:</u>
Implantation of EHM on dysfunctional left or right ventricular myocardium in patients with HFrEF (EF ≤35%).

Part A: Dose Finding Cohort to determine the Minimally Effective Dose and Optimally Effective Dose Range, and if possible the Safe Maximal Dose of EHM.

Part B: Refinement/Expansion Cohort to specify the most optimal EHM target heart wall, i.e. the left ventricle (LV) or the right ventricle (RV), and to collect proof-of-concept data as to efficacy of EHM mediated augmentation of the LV or RV by remuscularization.

Epicardial implantation will be via a minimal invasive left lateral thoracotomy performed as standalone procedure in case of LV targeting and concomitant to a scheduled open chest LV surgery if the RV is targeted. This strategy will reduce confounding effects as to the interpretation of EHM efficacy data.

<u>Duration of intervention per patient:</u>

Start of immune suppression 7±3 days before EHM implantation until the end of the study (daily intake of a calcineurin inhibitor and a corticosteroid for 12 months after EHM implantation)
Implantation of EHM: 90 minutes according to experience from preclinical studies and similar surgical procedures (i.e., epicardial pacemaker lead placement)

Note: After the final study visit (Visit 10), patients will be further monitored by their treating physician. Immune suppression by calcineurin inhibition will be continued until end-of-life if evidence for efficacy without safety concerns can be obtained within the 12 month study period. Corticoid administration will be stopped after 3-6 months according to guidelines for immune suppression in organ heart transplantation. The treating physician is requested to report clinically relevant observations to the principal investigator. After 12 month follow-up, study patients will be enrolled in a separate registry study (BioVAT-HF-registry set up by the Study Center) until end of life for the documentation of long-term outcome.
</td>
</tr>
</table>

	Follow-up per patient:<br>• 17 segment high-resolution echocardiography and MRI or CT to study global and regional heart/graft function (echo: before EHM implantation as well as 2 weeks, 1 month, 3 months, 6 months, and 12 months after surgery; MRI: before EHM implantation as well as 2 weeks, 3 months, 6 months, and 12 months after surgery; CT: before EHM implantation as well as 1 month and 12 months after surgery)<br>• Biomarkers: CK, CK-MB, cTnT, CRP, IL-6, and NT-proBNP; in addition, experimental assessment of graft derived DNA for the monitoring of graft retention/rejection (before EHM implantation as well as 2 weeks, 1, 3, 6, and 12 months after surgery)<br>• Telemetric monitoring via Implantable Cardioverter Defibrillator (ICD)- or Cardiac Resynchronization Therapy-Defibrillator (CRT-D)-devices with event recorder for the whole duration of the study.<br>• Pathology to obtain data on graft survival, integration, and maturation upon heart transplantation or death (according to patient consent).<br>• Monitoring of pulmonary artery pressure with a CardioMEMS HF Device (St. Jude Medical) or a similar device, if device has been implanted due to standard of care.<br><br>Accompanying measures:<br>• Therapeutic drug monitoring (TDM) to verify effective trough levels of accompanying immune suppressive drugs (calcineurin inhibitors) according to the proceeding in orthotopic heart transplantation (ISHLT Guidelines; Costanzo et al. 2010), i.e.:<br>for Tacrolimus:<br>10-15 ng/ml at the time of implantation maintained for 2 month followed by a reduction to 8-12 ng/ml until 6 months and finally a reduction to 5-10 ng/ml in stable patients<br>or alternatively<br>for Cyclosporine A:<br>275-375 ng/ml at the time of implantation maintained for 6 weeks followed by a reduction to 200-350 ng/ml until week 12 and then followed by a reduction to 150-300 ng/ml until 6 months and then followed by a further reduction to maintenance levels at 150-250 ng/ml.<br>• Biomarker analysis to monitor rejection: CK/CK-MB, cTnT, circulating cell-free allograft DNA (experimental method; substudy)<br>• Monitoring of specific allograft immune responses: donor specific antibodies (DSA)

**INCLUSION CRITERIA**	1. Heart failure with reduced ejection fraction (HFrEF with EF ≤ 35%) as assessed by high-resolution echocardiography and MRI or CT.<br>2. At least one hypo- or dyskinetic segment to demark the implant target area.<br>3. Stable disease condition allowing for an elective left-lateral mini-thoracotomy (for LV applications) or open-chest surgery (for RV applications) for a clinically indicated intervention on the LV (e.g., coronary bypass surgery, valve repair, mechanical circulatory support device implantation) with concomitant RV dysfunction, diagnosed using the Tricuspid Annular Plane Systolic Excursion (TAPSE) index <16 mm (Rudski et al. 2010).<br>4. 18-80 years of age<br>5. Previous implantation of an ICD or CRT-D with event recorder<br>6. New York Heart Association (NYHA) Class III or IV under optimal medical therapy<br>7. Willingness and ability to give written informed consent<br>8. Female subjects of childbearing potential must agree to use acceptable method(s) of contraception for the full study duration.
**EXCLUSION CRITERIA**	1. Contraindication to immunosuppressive drugs (e.g. known history of unresolved cancer, hepatitis B/C, HIV, HTLV1)<br>2. Contraindication to TachoSil® (e.g. hypersenstitivity to human fibrinogen, human thrombin, horse collagen, human albumin, Riboflavin, Natriumchloride, Natriumcitrate, L-Arginin-Hydrochloride)<br>3. Hypertrophic cardiomyopathy (HCM)<br>4. Terminal kidney failure (stage 4; GFR <30 ml/min) at the time of enrolment<br>5. Terminal liver failure (Child-Pugh stage C; score >10) at the time of enrolment<br>6. Autoimmune disease<br>7. History of disabling stroke<br>8. Reduced life expectancy in the short term due to non-cardiac disease<br>9. Any condition that excludes adherence to study protocol (in particular lack of adherence to prescribed medication)<br>10. Simultaneous participation in another interventional trial<br>11. Pregnant or breastfeeding females<br>12. Known or suspected alcohol and/or drug abuse

**SAFETY ENDPOINTS**	Assessment of safety:<br>Primary Safety Endpoint<br>• Part A (Dose Escalation steps): Adverse events related to the procedure, including in particular arrhythmic events and worsening of disease progression within 28 days (based on a comparison of data obtained during visit 2 and visit 7)<br>• Part B: Adverse events related to the procedure, including in particular arrhythmic events and worsening of disease progression within the whole study duration<br><br>Secondary Safety Endpoints:<br>• Frequency of major adverse cardiac events (MACE; non-fatal myocardial infarction, non-fatal stroke and cardiovascular death)<br>• Frequency and severity of arrhythmic events<br>• Incidence of immune rejection (allograft DNA, CK/CK-MB, cTnT. DSA)<br>• Incidence of mechanical perturbation of ventricular function by EHM graft
**EFFICACY ENDPOINTS**	Primary efficacy endpoint:<br>• Evidence for structural and functional muscular augmentation of target myocardium determined as enhanced target heart wall thickness (HWT) and thickening fraction (HWTF)<br><br>Key secondary endpoint:<br>• Recurrent HF hospitalizations<br><br>Further secondary endpoints:<br>• Left ventricular ejection fraction (EF)<br>• Change in heart failure medication<br>• Functional status in patients as determined by cardiopulmonary stress testing (VO2max), six-minute walk test (6MWT), and hand-grip strength measurements<br>• Patient reported outcomes assessed by NYHA classification, quality of life score (KCCQ, EQ-5D, QoL-VAD), and study adherence motivation (PHQ-9, HAF-17, ESSI, LOT-R, ULS-8, medication adherence, Trust/Mistrust in medical staff)<br>• All-cause and cardiovascular mortality
**TRIAL DESIGN**	Combined, open-label, phase I/II safety and efficacy study

**STATISTICAL ANALYSIS**	Primary Endpoints:<br>Primary efficacy analyses are based on the changes in HWT/HWTF between baseline (visit 2) and 2 weeks (visit 6), 1 month (visit 7), 3 months (visit 8), 6 months (visit 9) and 12 months (visit 10) after implantation. To test for a time effect a linear mixed model will be employed for each of the two primary endpoints.<br><br>Secondary endpoints:<br>Secondary endpoint analyses will be similar as the analyses of the primary endpoint and comprise of Gaussian longitudinal models evaluating changes over time from baseline prior to EHM implantation. For recurrent event data such as HF hospitalizations appropriate regression models such as the negative binomial regression model or the semiparametric LWYY model will be used.<br><br>Safety:<br>The maximal feasible dose (MFD; 20 g EHM comprised of 800 million cells) was chosen conservatively based on preclinical experience in Rhesus macaque and allometric scaling considerations. The probability of dose-limiting toxicity will be modelled by a Bayesian two-parameter logistic regression model. Safety events will be summarized as rates with 95% confidence intervals. Survival will be displayed as Kaplan-Meier curve and analyzed using a Cox proportional hazards model exploring the prognostic quality of the biomarkers assessed at baseline.	
	Effect size assumed for estimation of sample size:<br>A sample size of 30 patients (in Part B) yields a power of 80% (90%) in a pre-post comparison of means at a two-sided significance level of 10% given a standardized mean difference (Cohen's d) of 0.47 (0.55).	
**SAMPLE SIZE**	Part A:	n = 18 (max.), in dose cohorts of minimally 2 patients
	Part B	n=35 (min. 5 with LV and min. 5 with RV EHM placement; max. 30 per LV or RV indication)
	To be assessed for eligibility:	n = 65
	To be allocated to trial:	n = 53
	To be analysed:	n = 53
**TRIAL DURATION**	Time for preparation of the trial:	6 months
	Recruitment period (part A to part B):	60 months
	First patient in to last patient out (LPO):	72 months
	Post processing after LPO:	6 months
	Duration of the entire trial:	84 months

	Duration of surgical intervention per patient:	90 minutes (according to experience from preclinical studies and similar surgical procedures)
	Follow up duration per patient:	12 months
**PLANNED DATES**	Enrolment of first patient, first patient in (FPI)	1st quarter 2021
	Enrolment of last patient, last patient in (LPI)	4th quarter 2026
	End of trial defined as last patient last visit (LPLV)	4th quarter 2027
	Final statistical analysis	2nd quarter 2028
	Planned interim analysis	Interim analysis will be performed after end of study part A and after 15 of the patients treated with the SMD either with LV or RV EHM implantation have completed at least 3 month follow-up.
**PARTICIPATING SITES**	3 sites (Göttingen, Lübeck, Bad Oeynhausen) are planned in Germany.	
**FUNDER(S)**	The trial is funded by the DZHK *(Deutsches Zentrum für Herz-Kreislauf-Forschung e.V.)* and Repairon GmbH.	

Supplementary Data (refer to excel spreadsheets)

Supplementary Data 1: Therapeutic Drug Monitoring - Calcineurin Inhibitors

Supplementary Data 2: Donor-specific antibodies (DSA)

Supplementary Data 3: Clinical Chemistry

Supplementary Videos:

Supplementary Video 1: Contracting Rhesus EHM suspended in Ringer solution. Spontaneous contractions of EHM can be readily observed.

Supplementary Video 2: MRI documentation 2 months after EHM implantation in a healthy Rhesus macaque (#2444). Refer to **Figure 2** for a still image with arrows indicating the EHM graft.

Supplementary Video 3: Mechanically triggered contraction in human EHM. Ring-shaped human EHM 1 (mechanically stimulated) and EHM 2 (spontaneously contracting/not mechanically stimulated) suspended on flexible poles of an EHM patch holding device. Recordings were performed at room temperature.
