# Supplementary material for: Engineered heart muscle allografts for heart repair in primates and humans
Source: Nature. 2025 Jan 29;639(8054):503–11. doi: 10.1038/s41586-024-08463-0 (PMC11903342; doi:10.1038/s41586-024-08463-0)
Supplement: Supplementary file 2 — Reporting Summary [file 41586_2024_8463_MOESM2_ESM.pdf]

nature portfolio

Corresponding author(s): Wolfram-H. Zimmermann

Last updated by author(s): Oct 29, 2024

Reporting Summary

Nature Portfolio wishes to improve the reproducibility of the work that we publish. This form provides structure for consistency and transparency in reporting. For further information on Nature Portfolio policies, see our Editorial Policies and the Editorial Policy Checklist.

Statistics

For all statistical analyses, confirm that the following items are present in the figure legend, table legend, main text, or Methods section.

n/a	Confirmed	
☐	☒	The exact sample size (n) for each experimental group/condition, given as a discrete number and unit of measurement
☐	☒	A statement on whether measurements were taken from distinct samples or whether the same sample was measured repeatedly
☐	☒	The statistical test(s) used AND whether they are one- or two-sided<br>*Only common tests should be described solely by name; describe more complex techniques in the Methods section.*
☒	☐	A description of all covariates tested
☐	☒	A description of any assumptions or corrections, such as tests of normality and adjustment for multiple comparisons
☐	☒	A full description of the statistical parameters including central tendency (e.g. means) or other basic estimates (e.g. regression coefficient) AND variation (e.g. standard deviation) or associated estimates of uncertainty (e.g. confidence intervals)
☐	☒	For null hypothesis testing, the test statistic (e.g. F, t, r) with confidence intervals, effect sizes, degrees of freedom and P value noted<br>*Give P values as exact values whenever suitable.*
☒	☐	For Bayesian analysis, information on the choice of priors and Markov chain Monte Carlo settings
☒	☐	For hierarchical and complex designs, identification of the appropriate level for tests and full reporting of outcomes
☒	☐	Estimates of effect sizes (e.g. Cohen's d, Pearson's r), indicating how they were calculated

Our web collection on statistics for biologists contains articles on many of the points above.

Software and code

Policy information about availability of computer code

Data collection	BD FACSDiva Software (Version 6.1.3), Kaluza 2.2.1 (Beckman), VitroDat 3.52 (Föhr Medical Instruments), Zen 2.3 SP1 (Zeiss)
Data analysis	Microsoft Excel 2019 MSO (16.0.10415.20025) 32-Bit, Graph Pad Prism 10.1.2 (64-bit), FlowJo 9.6 software, BD FACSDiva Software (Version 6.1.3), Kaluza 2.2.1 (Beckman), CellRanger (v.3.1.0; 10XGenomics), SeqPilot (JSI medical systems GmbH, Version 5.2.0 Build 505), Varbank 2.0 (Cologne Center for Genomics (CCG); University of Cologne), VitroDat 3.52 (Föhr Medical Instruments), Zen 2.3 SP1 (Zeiss), Segment v4.0 R12067 (Medviso, segment.heiberg.se), Medis-Suite Version 3.2 with QMass module Version 8.1 (Medis)

For manuscripts utilizing custom algorithms or software that are central to the research but not yet described in published literature, software must be made available to editors and reviewers. We strongly encourage code deposition in a community repository (e.g. GitHub). See the Nature Portfolio guidelines for submitting code & software for further information.

Data

Policy information about availability of data

All manuscripts must include a data availability statement. This statement should provide the following information, where applicable:

- Accession codes, unique identifiers, or web links for publicly available datasets
- A description of any restrictions on data availability
- For clinical datasets or third party data, please ensure that the statement adheres to our policy

The data sets generated during and/or analysed during the current study are available from the corresponding author. Please refer also to the Source Data

document. The snRNAseq data is publicly accessible under GSE276021 (GEO data base). Gene counts were obtained by aligning reads to the hg38 genome (NCBI:GCA_000001405.22; GRCh38.p7) using CellRanger software (v.3.0.2; 10XGenomics).

Human research participants

Policy information about studies involving human research participants and Sex and Gender in Research.

Reporting on sex and gender	gender is reported
Population characteristics	not appliacable
Recruitment	Case report from BioVAT-HF-DZHK20 - EudraCT No. 2019-000885-39 [EU CT No. 2024-515708-38-01] and ClinicalTrials.gov ID NCT04396899
Ethics oversight	The BioVAT-HF-DZHK20 Phase I/II clinical trial (ClinicalTrial.gov NCT04396899) was approved by the responsible regulatory agency (Paul-Ehrlich-Institute) and the competent ethics committee (ethics committee of the University Medical Center Göttingen under the file #18/7/20).

Note that full information on the approval of the study protocol must also be provided in the manuscript.

Field-specific reporting

Please select the one below that is the best fit for your research. If you are not sure, read the appropriate sections before making your selection.

☒ Life sciences ☐ Behavioural & social sciences ☐ Ecological, evolutionary & environmental sciences

For a reference copy of the document with all sections, see nature.com/documents/nr-reporting-summary-flat.pdf

Life sciences study design

All studies must disclose on these points even when the disclosure is negative.

Sample size	Sample size (n=7 Cohort 1 ; n=7 in Cohort 2; n=20 in Cohort 3) was chosen based on previous experience and taking in account 3R considerations. The adaptive study design in Cohorts 1 and 2 informed choice of immune suppression and dosing in Cohort 3. Refer to Supplementary Table 3 for an overview of all Rhesus macaques included in the study.
Data exclusions	No data was excluded
Replication	Data was replicated in Cohorts 1 to 3 with adequate groups sizes of 7 (Cohort 1), 7 (Cohort 2) and 20 (Cohort 3 - 1 animal was not allowed to be included in the implantation study due to low body weight, 5 animals died post myocardial infarction, 1 animal died upon weaning from anesthesia after implantation of a 5x EHM).
Randomization	Rat study: animals were assigned radomly to the experiemtal groups (with vital or irradiated EHM implant). Rhesus macaque study: allograft animals were assigned to the different study groups by coin flip.
Blinding	With the exception of the surgeons (blinding is not possible), investigators were blinded to the study protocol. Recording of MRI data were performed by investigators blinded to the treatment condition. MRI image analysis of Cohorts 1 and 2 were performed by 2 independent observers. Investigations of MRI data of Cohorts 3 were performed by 2 additional independent observers. Pathological analyses were performed by as to the treatment condition blinded pathologists.

Behavioural & social sciences study design

All studies must disclose on these points even when the disclosure is negative.

Study description	*Briefly describe the study type including whether data are quantitative, qualitative, or mixed-methods (e.g. qualitative cross-sectional, quantitative experimental, mixed-methods case study).*
Research sample	*State the research sample (e.g. Harvard university undergraduates, villagers in rural India) and provide relevant demographic information (e.g. age, sex) and indicate whether the sample is representative. Provide a rationale for the study sample chosen. For studies involving existing datasets, please describe the dataset and source.*
Sampling strategy	*Describe the sampling procedure (e.g. random, snowball, stratified, convenience). Describe the statistical methods that were used to predetermine sample size OR if no sample-size calculation was performed, describe how sample sizes were chosen and provide a rationale for why these sample sizes are sufficient. For qualitative data, please indicate whether data saturation was considered, and what criteria were used to decide that no further sampling was needed.*

Data collection	*Provide details about the data collection procedure, including the instruments or devices used to record the data (e.g. pen and paper, computer, eye tracker, video or audio equipment) whether anyone was present besides the participant(s) and the researcher, and whether the researcher was blind to experimental condition and/or the study hypothesis during data collection.*
Timing	*Indicate the start and stop dates of data collection. If there is a gap between collection periods, state the dates for each sample cohort.*
Data exclusions	*If no data were excluded from the analyses, state so OR if data were excluded, provide the exact number of exclusions and the rationale behind them, indicating whether exclusion criteria were pre-established.*
Non-participation	*State how many participants dropped out/declined participation and the reason(s) given OR provide response rate OR state that no participants dropped out/declined participation.*
Randomization	*If participants were not allocated into experimental groups, state so OR describe how participants were allocated to groups, and if allocation was not random, describe how covariates were controlled.*

Ecological, evolutionary & environmental sciences study design

All studies must disclose on these points even when the disclosure is negative.

Study description	*Briefly describe the study. For quantitative data include treatment factors and interactions, design structure (e.g. factorial, nested, hierarchical), nature and number of experimental units and replicates.*
Research sample	*Describe the research sample (e.g. a group of tagged Passer domesticus, all Stenocereus thurberi within Organ Pipe Cactus National Monument), and provide a rationale for the sample choice. When relevant, describe the organism taxa, source, sex, age range and any manipulations. State what population the sample is meant to represent when applicable. For studies involving existing datasets, describe the data and its source.*
Sampling strategy	*Note the sampling procedure. Describe the statistical methods that were used to predetermine sample size OR if no sample-size calculation was performed, describe how sample sizes were chosen and provide a rationale for why these sample sizes are sufficient.*
Data collection	*Describe the data collection procedure, including who recorded the data and how.*
Timing and spatial scale	*Indicate the start and stop dates of data collection, noting the frequency and periodicity of sampling and providing a rationale for these choices. If there is a gap between collection periods, state the dates for each sample cohort. Specify the spatial scale from which the data are taken*
Data exclusions	*If no data were excluded from the analyses, state so OR if data were excluded, describe the exclusions and the rationale behind them, indicating whether exclusion criteria were pre-established.*
Reproducibility	*Describe the measures taken to verify the reproducibility of experimental findings. For each experiment, note whether any attempts to repeat the experiment failed OR state that all attempts to repeat the experiment were successful.*
Randomization	*Describe how samples/organisms/participants were allocated into groups. If allocation was not random, describe how covariates were controlled. If this is not relevant to your study, explain why.*
Blinding	*Describe the extent of blinding used during data acquisition and analysis. If blinding was not possible, describe why OR explain why blinding was not relevant to your study.*

Did the study involve field work? ☐ Yes ☐ No

Field work, collection and transport

Field conditions	*Describe the study conditions for field work, providing relevant parameters (e.g. temperature, rainfall).*
Location	*State the location of the sampling or experiment, providing relevant parameters (e.g. latitude and longitude, elevation, water depth).*
Access & import/export	*Describe the efforts you have made to access habitats and to collect and import/export your samples in a responsible manner and in compliance with local, national and international laws, noting any permits that were obtained (give the name of the issuing authority, the date of issue, and any identifying information).*
Disturbance	*Describe any disturbance caused by the study and how it was minimized.*

Reporting for specific materials, systems and methods

We require information from authors about some types of materials, experimental systems and methods used in many studies. Here, indicate whether each material, system or method listed is relevant to your study. If you are not sure if a list item applies to your research, read the appropriate section before selecting a response.

Materials & experimental systems

n/a	Involved in the study
☐	☒ Antibodies
☐	☒ Eukaryotic cell lines
☒	☐ Palaeontology and archaeology
☐	☒ Animals and other organisms
☐	☒ Clinical data
☒	☐ Dual use research of concern

Methods

n/a	Involved in the study
☒	☐ ChIP-seq
☐	☒ Flow cytometry
☒	☐ MRI-based neuroimaging

Antibodies

Antibodies used

Refer to Supplementary Table 6 for details

Validation

Antibodies used in this study were validated in previous studies (e.g., Riegler et al. 2015 Circ Res, Tiburcy et al. 2017 Circulation). Antibodies used in the pathology studies are all validated for veterinarian and clinical use and tested for cross-reactivity to Rhesus macaque samples.

Eukaryotic cell lines

Policy information about cell lines and Sex and Gender in Research

Cell line source(s)

Rhesus iPSC-lines:
iPSC 43110-4: fibroblast obtained from the California National Primate Research Center in Davis were reprogrammed using Sendai Virus mediated deliver of Oct4, Sox2, Nanog, and cMyc as reported in Zhao et al 2018 (https://doi.org/10.1016/j.stemcr.2018.01.002) and Yang et al. 2021 (https://doi.org/10.1093/cvr/cvaa281)

DPZ_iRH34.1 and DPZ_iRH23.1 (from animal #2500 - autograft recipient): fibroblast obtained from the Deutsches Primatenzentrum (German Primate Center) in Göttingen were reprogrammed by nucleofection of three episomal vectors pCXLE-hOCT3/4-shp53-F (encoding for human OCT3/4 and shRNA against p53; Addgene #27077), pCXLE-hSK (encoding for human SOX2 and KLF4; Addgene #27078) and pCXLE-hUL (encoding for human L-MYC and LIN28; Addgene #27080) as reported in Stauske et al. 2020 (doi:10.3390/cells9061349).

DPZ_iRH25.B1 (from animal #2483 - autograft recipient): fibroblast obtained from the Deutsches Primatenzentrum (German Primate Center) were reprogrammed using Sendai Virus mediated delivery of Oct4, Sox2, Nanog, and cMyc similar as described in Zhao et al 2018 (https://doi.org/10.1016/j.stemcr.2018.01.002) and Yang et al. 2021 (https://doi.org/10.1093/cvr/cvaa281).

Human iPSC-line:
Generation of the GMP line LiPSC-GR1.1 (also referred to as TC1133; lot number 50-001-21) was supported by the NIH Common Fund Regenerative Medicine Program, and reported in Baghbaderani et al. 2015 (doi:10.1016/j.stemcr.2015.08.015). Further information is available at https://hpscreg.eu/cell-line/RUCDRi002-A. The NIH Common Fund and the National Center for Advancing Translational Sciences (NCATS) are joint stewards of the LiPSC-GR1.1 resource. A derivative from a GMP master cell bank of the TC1133-line was obtained by Repairon GmbH and was used as starting material for EHM production for the BioVAT-HF-DZHK20 Phase I/II clinical trial.

Authentication

By MHC (human) or Mamu (macaque) typing

Mycoplasma contamination

All lines were tested to be free from mycoplasma contamination (in-house testing using Lonza MycoAlert® Detection Kit, external testing of GMP cell lines and products by Minerva Biolabs, Berlin, Germany).

Commonly misidentified lines
(See ICLAC register)

no commonly misidentified cell lines were used in the study

Palaeontology and Archaeology

Specimen provenance

Provide provenance information for specimens and describe permits that were obtained for the work (including the name of the issuing authority, the date of issue, and any identifying information). Permits should encompass collection and, where applicable, export.

Specimen deposition	*Indicate where the specimens have been deposited to permit free access by other researchers.*
Dating methods	*If new dates are provided, describe how they were obtained (e.g. collection, storage, sample pretreatment and measurement), where they were obtained (i.e. lab name), the calibration program and the protocol for quality assurance OR state that no new dates are provided.*

☐ Tick this box to confirm that the raw and calibrated dates are available in the paper or in Supplementary Information.

Ethics oversight	*Identify the organization(s) that approved or provided guidance on the study protocol, OR state that no ethical approval or guidance was required and explain why not.*

Note that full information on the approval of the study protocol must also be provided in the manuscript.

Animals and other research organisms

Policy information about studies involving animals; ARRIVE guidelines recommended for reporting animal research, and Sex and Gender in Research

Laboratory animals	Rats: 8-10 weeks old male RNU rats (250-350g) obtained from Charles River Laboratories, Wilmington, MA<br>Rhesus macaques: refer to Supplementary Table 3 for a detailed overview of the experimental animals used in this study.
Wild animals	No wild animals were used in the study.
Reporting on sex	refer to Supplementary Table 3 for a detailed overview of the experimental animals (Macaca mulatta) used in this study
Field-collected samples	No field collected samples were used in the study.
Ethics oversight	Animal experiments were approved by the by the Stanford Animal Research Committee (nude rat study at Stanford) and Niedersächsisches Landesamt für Verbraucherschutz und Lebensmittelsicherheit (LAVES; 33.42502-04-15/1807 and -16/2370; Rhesus macaque [Macaca mulatta] studies in Göttingen).

Note that full information on the approval of the study protocol must also be provided in the manuscript.

Clinical data

Policy information about clinical studies

All manuscripts should comply with the ICMJE guidelines for publication of clinical research and a completed CONSORT checklist must be included with all submissions.

Clinical trial registration	EU CT No. 2024-515708-38-01 (previously EudraCT: 2019-000885-39) and ClinicalTrials.gov: NCT04396899
Study protocol	We are reporting a case from the BioVAT-HF-DZHK20 Phase I/II clinical trial. Refer to Supplementary Note 5 for Clinical Reserach Information and the Synopsis of the BioVAT-HF-DZHK20 Clinical Trial Protocol.
Data collection	Here we are reporting immunohistochemistry and clinical data from a heart explant patient included in the BioVAT-HF-DZHK20 Phase I/II clinical trial (case report). The release of the data was approved by the Sponsor (University Medical Center Göttingen) and the repsonsible clinical trial statistician (Prof. T. Friede, Institut of Medical Statistics). The clinical trial is ongoing and the full data set will be reported seperately after completion of the clinical trial.
Outcomes	iPSC-derived cardiomyocyte allograft retention was determined by histopathology (H&E staining and immunohistochemistry) and deep sequencing of microdissected FFPE-samples followed by sequence alignment to determine host and graft specific single nucleotide variants (SNVs).

Dual use research of concern

Policy information about dual use research of concern

Hazards

Could the accidental, deliberate or reckless misuse of agents or technologies generated in the work, or the application of information presented in the manuscript, pose a threat to:

No	Yes	
☒	☐	Public health
☒	☐	National security
☒	☐	Crops and/or livestock
☒	☐	Ecosystems
☒	☐	Any other significant area

Experiments of concern

Does the work involve any of these experiments of concern:

No	Yes	
☒	☐	Demonstrate how to render a vaccine ineffective
☒	☐	Confer resistance to therapeutically useful antibiotics or antiviral agents
☒	☐	Enhance the virulence of a pathogen or render a nonpathogen virulent
☒	☐	Increase transmissibility of a pathogen
☒	☐	Alter the host range of a pathogen
☒	☐	Enable evasion of diagnostic/detection modalities
☒	☐	Enable the weaponization of a biological agent or toxin
☒	☐	Any other potentially harmful combination of experiments and agents

ChIP-seq

Data deposition

☐ Confirm that both raw and final processed data have been deposited in a public database such as GEO.

☐ Confirm that you have deposited or provided access to graph files (e.g. BED files) for the called peaks.

Data access links<br>*May remain private before publication.*	*For "Initial submission" or "Revised version" documents, provide reviewer access links. For your "Final submission" document, provide a link to the deposited data.*
Files in database submission	*Provide a list of all files available in the database submission.*
Genome browser session<br>(e.g. UCSC)	*Provide a link to an anonymized genome browser session for "Initial submission" and "Revised version" documents only, to enable peer review. Write "no longer applicable" for "Final submission" documents.*

Methodology

Replicates	*Describe the experimental replicates, specifying number, type and replicate agreement.*
Sequencing depth	*Describe the sequencing depth for each experiment, providing the total number of reads, uniquely mapped reads, length of reads and whether they were paired- or single-end.*
Antibodies	*Describe the antibodies used for the ChIP-seq experiments; as applicable, provide supplier name, catalog number, clone name, and lot number.*
Peak calling parameters	*Specify the command line program and parameters used for read mapping and peak calling, including the ChIP, control and index files used.*
Data quality	*Describe the methods used to ensure data quality in full detail, including how many peaks are at FDR 5% and above 5-fold enrichment.*
Software	*Describe the software used to collect and analyze the ChIP-seq data. For custom code that has been deposited into a community repository, provide accession details.*

Flow Cytometry

Plots

Confirm that:

☒ The axis labels state the marker and fluorochrome used (e.g. CD4-FITC).

☒ The axis scales are clearly visible. Include numbers along axes only for bottom left plot of group (a 'group' is an analysis of identical markers).

☒ All plots are contour plots with outliers or pseudocolor plots.

☒ A numerical value for number of cells or percentage (with statistics) is provided.

Methodology

Sample preparation	Flow cytomry analysis of EHM cell composition. EHM were washed in PBS and dissociated in 2 mg/ml Collagenase 1 (Sigma-Aldrich) in PBS with 20% FBS at 37°C for 1 h followed by Accutase (Millipore), 0.025% Trypsin (ThermoScientific) and 20 µg/ml DNAse I (Calbiochem) at 20-24°C for 30 min. After fixation in 70% ice cold EtOH for >10 min, cells were either exposed to primary antibody directed against sarcomeric actinin (ACTN2: 1:4,000; A7811, Sigma) or vimentin (VIM; 1:1,000; ab92547, abcam) in blocking buffer for 45 min followed by secondary antibodies in blocking buffer and Hoechst 33342 for 30 min at 4°

C (Supplementary Table 6). Control samples were exposed to undirected IgG1 (MAB002; R&D Systems). Human samples were fixed with 4% formalin and exposed to conjugated antibodies directed against sarcomeric actinin (ACTN2-PE, 1:1000, 130-106-937, Miltenyi Biotec) and vimentin (VIM-AF647, 1:1000, Biolegend, 677807) for 15 min at 4°C. A BD LSRII SORP system (BD Biosciences) or CytoFLEX (Beckman/Coulter) was used for flow cytometry analysis.

Donor-specific antibody analysis. iPSC-derived cardiomyocytes (CMs) and stromal cells (StCs), unstimulated and after IFNγ (100 ng/mL for 48 h) were exposed to sera obtained before (pre) and at the indicated timepoints during the study at different dilutions (1:5 to 1:40). A FITC-labeled anti-Rhesus IgG antibody (4700-02, Southern Biotech; Birmingham, AL, USA) was used to detect antibodies in the sera bound to the CMs and StCs. The cell mean fluorescence intensity (MFI) and the proportion of stained cells were determined by flow cytometry (LSR II SORP, BD Biosciences). Antibodies that display a selective reactivity to IFNγ stimulated CMs presumably include DSAs to MHC class I molecules. The pan-HLA antibody W6/32 (Biolegend, San Diego, CA, USA), which reacts with MHC class I molecules of rhesus macaques was used to demonstrate the expression of these molecules on CMs and StCs.

Flow Cytometry analysis of peripheral immune cells. 50 µl of whole blood were stained with a mixture of pre-titrated monoclonal antibodies (refer to antibody information in Supplementary Table 6) for 30 min at room temperature in the dark. Lysis of red blood cells and fixation was performed by incubation with 1 ml RBC lysis/fixation solution (BioLegend, San Diego, CA) for 15 minutes. Following a washing step with PBS/BSA cells were analyzed using a LSRII cytometer (BD Biosciences) and FlowJo 9.6 software (Treestar, Ashland, OR).

Instrument

BD LSRII SORP system (BD Biosciences)

Software

FlowJo 9.6 software, BD FACSDiva Software, Kaluza 2.2 (Beckman)

Cell population abundance

Cell sorting was not applied

Gating strategy

Gating strategy for cardiomyocyte and stromal cell quantification: Living cells were gated based on nuclear DNA signal after labeling with Hoechst-33342 (Pacific Blue-channel). Single cells were separated from cell aggregates. Cardiomyocytes and stromal cells were either labeled with antibodies directed against ACTN2 or VIM, respectively, and detected with an Alexa Flour-488 (FITC-channel) conjugated secondary antibody or exposed to fluorochrome-conjugated antibodies.

Gating strategy for donor specific antibody (DSA) detection: Cardiomyocytes (CM) not stimulated or stimulated with IFN-γ for 48 h were gated based on FSC-A and SSC-A parameters to exclude debris and not incubated or incubated with 1:20 diluted sera obtained from #2887 16 weeks after EHM implantation and after withdrawal of immunosuppression. 20.000 events were measured. A FITClabeled anti-rhesus IgG antibody detected antibodies in the sera bound to the CMs. In addition to the mean fluorescence intensity, the proportion of stained CMs has been determined using the second marker (FITC-A subset 2). The expression of MHC class I molecules on the CMs used in this experiment was determined in parallel using the W6/32 antibody and a FITC-labeled secondary antibody against mouse IgG. Antibodies that display a selective reactivity to IFN-γ-stimulated CMs presumably include DSAs to MHC class I molecules.

Gating strategy peripheral blood mononuclear cells: Leukocytes were gated based on CD45 expression versus SSC-A. Following exclusion of douplets either CD11c+ cells or CD45+ lymphocytes were further gated. T and B cells were distinguished based on CD3 versus CD20 expression. T cells were further divided into CD4+ and CD8+ T cells. NK cells were identified as CD3- CD20-/CD8+ CD159a+ cells. Activation of immune cells was assessed by analyzing CD80 expression on B cells and CD11c+ cells as well CD69 and HLA-DR expression on total CD3+, CD4+, CD8+ T cells and NK cells.

Refer for further details to Supplementary Information: Supplementary Method 1_Flow Cytometry Gating Strategy

☒ Tick this box to confirm that a figure exemplifying the gating strategy is provided in the Supplementary Information.

Magnetic resonance imaging

Experimental design

Design type

Indicate task or resting state; event-related or block design.

Design specifications

Specify the number of blocks, trials or experimental units per session and/or subject, and specify the length of each trial or block (if trials are blocked) and interval between trials.

Behavioral performance measures

State number and/or type of variables recorded (e.g. correct button press, response time) and what statistics were used to establish that the subjects were performing the task as expected (e.g. mean, range, and/or standard deviation across subjects).

Acquisition

Imaging type(s) | *Specify: functional, structural, diffusion, perfusion.*

Field strength | *Specify in Tesla*

Sequence & imaging parameters | *Specify the pulse sequence type (gradient echo, spin echo, etc.), imaging type (EPI, spiral, etc.), field of view, matrix size, slice thickness, orientation and TE/TR/flip angle.*

Area of acquisition | *State whether a whole brain scan was used OR define the area of acquisition, describing how the region was determined.*

Diffusion MRI ☐ Used ☐ Not used

Preprocessing

Preprocessing software | *Provide detail on software version and revision number and on specific parameters (model/functions, brain extraction, segmentation, smoothing kernel size, etc.).*

Normalization | *If data were normalized/standardized, describe the approach(es): specify linear or non-linear and define image types used for transformation OR indicate that data were not normalized and explain rationale for lack of normalization.*

Normalization template | *Describe the template used for normalization/transformation, specifying subject space or group standardized space (e.g. original Talairach, MNI305, ICBM152) OR indicate that the data were not normalized.*

Noise and artifact removal | *Describe your procedure(s) for artifact and structured noise removal, specifying motion parameters, tissue signals and physiological signals (heart rate, respiration).*

Volume censoring | *Define your software and/or method and criteria for volume censoring, and state the extent of such censoring.*

Statistical modeling & inference

Model type and settings | *Specify type (mass univariate, multivariate, RSA, predictive, etc.) and describe essential details of the model at the first and second levels (e.g. fixed, random or mixed effects; drift or auto-correlation).*

Effect(s) tested | *Define precise effect in terms of the task or stimulus conditions instead of psychological concepts and indicate whether ANOVA or factorial designs were used.*

Specify type of analysis: ☐ Whole brain ☐ ROI-based ☐ Both

Statistic type for inference
(See Eklund et al. 2016) | *Specify voxel-wise or cluster-wise and report all relevant parameters for cluster-wise methods.*

Correction | *Describe the type of correction and how it is obtained for multiple comparisons (e.g. FWE, FDR, permutation or Monte Carlo).*

Models & analysis

n/a	Involved in the study
☐	☐ Functional and/or effective connectivity
☐	☐ Graph analysis
☐	☐ Multivariate modeling or predictive analysis

Functional and/or effective connectivity | *Report the measures of dependence used and the model details (e.g. Pearson correlation, partial correlation, mutual information).*

Graph analysis | *Report the dependent variable and connectivity measure, specifying weighted graph or binarized graph, subject- or group-level, and the global and/or node summaries used (e.g. clustering coefficient, efficiency, etc.).*

Multivariate modeling and predictive analysis | *Specify independent variables, features extraction and dimension reduction, model, training and evaluation metrics.*
